# Were public interventions relevant for containing the covid-19 pandemic in Brazil in 2020?

**DOI:** 10.11606/s1518-8787.2023057005030

**Published:** 2023-10-24

**Authors:** Vitória Berg Cattani, Thaís Araujo dos Santos, Marcelo Ribeiro-Alves, Julio Castro-Alves

**Affiliations:** I Fundação Oswaldo Cruz Instituto Nacional de Infectologia Evandro Chagas Rio de Janeiro RJ Brasil Fundação Oswaldo Cruz . Instituto Nacional de Infectologia Evandro Chagas . Rio de Janeiro , RJ , Brasil .

**Keywords:** Public Policy, Mortality, SARS-CoV-2, COVID-19, Communicable Disease Control

## Abstract

**OBJECTIVE:**

Flattening the curve was the most promoted public health strategy worldwide, during the pandemic, to slow down the spread of the SARS-CoV-2 virus, and, consequently, to avoid overloading the healthcare systems. In Brazil, a relative success of public policies was evidenced. However, the association between public policies and the “flatten the curve” objectives remain unclear, as well as the association of different policies to reach this aim. This study aims to verify if the adoption of different public policies was associated with the flattening of the infection and death curves by covid-19 first wave in 2020.

**METHODS:**

Data from the Sistema de Informação da Vigilância Epidemiológica da Gripe (Influenza Epidemiological Surveillance Information System – SIVEP-Gripe) and the Instituto Brasileiro de Geografia e Estatística (Brazilian Institute of Geography and Statistics – IBGE) were used to compute standardized incidence and mortality rates. The Oxford Covid-19 Government Response Tracker (OxCGRT) was used to obtain information about governmental responses related to the mitigation of pandemic effects, and the Human Development Index (HDI) was used as a measure of socioeconomic status. A non-linear least-square method was used to estimate parameters of the five-parameter sigmoidal curve, obtaining the time to reach the peak and the incremental rate of the curves. Additionally, ordinary least-square linear models were used to assess the correlation between the curves and the public policies adopted.

**RESULTS:**

Out of 51 municipalities, 261,326 patients had SARS-CoV-2 infection. Stringency Index was associated with reducing covid-19 incremental incidence and death rates,in addition to delaying the time to reach the peak of both pandemic curves. Considering both parameters, economic support policies did not affect the incidence nor the mortality rate curves.

**CONCLUSION:**

The evidence highlighted the importance and effectiveness of social distancing policies during the first year of the pandemic in Brazil, flattening the curves of mortality and incidence rates. Other policies, such as those focused on economic support, were not effective in flattening the curves but met humanitarian and social outcomes.

## INTRODUCTION

The novel coronavirus (SARS-CoV-2) was identified in December 2019. The virus had a rapid spread ^1^ , with 15% of infections progressing to severe infections, thus requiring hospitalization. Additionally, 5% of cases were critical infections ^2^ , which demanded hospitalization in intensive care medical units (ICU) and use of mechanical ventilation.

Due to the high demand for healthcare services, the most promoted public health worldwide strategy during the pandemic was to “flatten the curve.” Flattening the curve means slowing the spread of the epidemic so that the peak number of people requiring care at a time is reduced, and the time to reach this peak is delayed. Mandatory public policies were implemented to influence the population’s mobility pattern and contributed to increasing adherence to social distancing.

In Brazil—a dramatic case of failure in mitigating the consequences of the pandemic—public policies did not have centralized coordination between levels of government, causing heterogeneity of time and intensity in the promotion of public policies aimed at flattening the curves. Barberia ^3^ , using Oxford Covid-19 Government Response Tracker (OxCGRT) ^4^ , verified that sub-national governments, especially state governments, played an important role in implementing social distancing measures to halt the spread of the virus when compared to the federal government. The negative effect of the federal government, headed by a president who minimized the disease’s consequences and disregarded the importance of social distancing, was also extensively documented ^5 , 6^ . However, evidence have shown that mandatory policies on social distancing increased the levels of adherence ^7 , 8^ , particularly when a more complete and rigorous set of policies was adopted ^9^ . Furthermore, the population adherence to social distancing was associated with a decrease in the mortality rate ^10 , 11^ . Despite the evidence on the relative success of public policies ^12^ , other aspects demand clarification, such as (i) the association of public policies with the incidence and mortality rate of hospitalization by SARS-CoV-2 infections; (ii) the association of public policies with reducing the peak incidence of people requiring care and delaying the time to reach this peak (“flatten the curve” goal); and, (iii) the success of different policies to reach these goals.

This study aims to verify if the adoption of different public policies, such as containment and closure, economic support, health services, and testing, were associated with the flattening of the infection and death curves by covid-19 first wave, from March to December 2020, in the Brazilian hospitals.

## METHODS

### Data

#### Health Data

The information on the number of SARS-CoV-2 infections, deaths, the pandemic time (date of first symptoms for the first recorded infection by the municipality of residence), sex, and age were extracted from the *Sistema de Informação da Vigilância Epidemiológica da Gripe* (Influenza Epidemiological Surveillance Information System – SIVEP-Gripe), as of January 11, 2021 (downloaded from https://opendatasus.saude.gov.br/dataset/srag-2020). Then, patients were only selected in cases with a confirmed diagnosis of covid-19 (laboratory, clinical, clinical-epidemiological, or imaging criteria) notified from February 20 (the first confirmed infection of covid-19 in Brazilian territory) to December 31, 2020. Patients with ongoing clinical evolution on December 31, 2020, were excluded.

#### Public Policies

Daily data from The Oxford covid-19 Government Response Tracker (OxCGRT) on several different common governments’ policy responses ^4^ were collected from 55 Brazilian municipalities from January 1, 2020, to December 31, 2020 (downloaded from https://github.com/OxCGRT/Brazil-covid-policy). Indexes that describe the overall policy environment that applies to residents of the city were used, including the policies set by either the national or state governments. Among them, the Stringency Index was used, which includes containment and closure policies, such as the closings of schools, universities, workplaces, and public transport (including intercity transport); canceling of public events; limiting of private gatherings; directives to “shelter-in-place” and confine at home; restricting of movement between cities, regions, and countries; and the promoting of public information campaigns. We also used the Economic Support Index, which contains income support and debt/contract relief policies to households. Containment and Health Index, that represents all policies included in the Stringency Index augmented by health policies, such as testing policy, contact tracing, facial coverings, vaccination policy, and protection of older adults. Finally, the Government Response Index, that combines the Containment and Health Index and the Economic Support Index. All these daily index data were aggregated into the corresponding time-span period for each municipality.

#### Adjustment Variables

To control socioeconomic discrepancies between municipalities, the Human Development Index (HDI) was included as a measure of socioeconomic status. The aggregate HDI, as well as its three dimensions, is represented by a number ranging from 0 to 1; the closer to 1, the greater the human development of the population. To control for environmental causes of airborne transmission among people, the Crowding Rate (percentage of the population living in a house with more than two people per room in 2010) and the Demographic Density Rate (number of inhabitants per km ^2^ in 2010) were included. To control the discrepancies in healthcare assessment between municipalities, the ICU Hospital Beds Rate (number of hospital beds in ICU per million inhabitants in 2015) and the Physician Rate (number of physicians per thousand inhabitants in 2015) were included.

## Statistical Analysis

**Figure 1:**
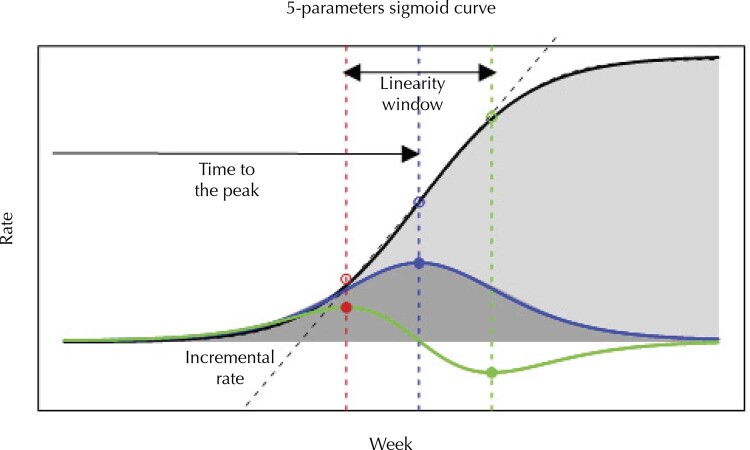
Illustration of curves generated by hypothetical rate to estimate the parameters, time to the peak and incremental rate, using a 5-parameters curve model.

Note: Absolute (dark gray) and cumulative (light gray) case/death rates, and first (solid blue) and second (solid green) derivative curves of the latter. The incremental rate (dashed black line) indicates the growth rate during the “window of linearity,” which is the largest linearity region of the cumulative curve. The boundaries for this region are points (red and green rings) corresponding to the maximum (red circle/dashed red line) and minimum (green circle/dashed green line) points of the second derivate curve. The time to reach the peak of the absolute rate curve is the corresponding time inside the “window of linearity” where the second derivate is zero (dashed blue line), or, in other words, where the rate start to decline in a municipality.

Standardized incidence and mortality rates (per 100,000 people) were computed, calculated as the number of infections and the number of deaths related to covid-19 by the total population exposed to the risk, respectively. Rates were standardized by age using the 2010 Brazilian Population Pyramid (age and sex pyramid) as a reference. The standardized incidence rate (SIR) included three distinct time-spans to consider the exact day of infection, that is, 5, 9, or 13 days before the date of registration at the SIVEP-Gripe/MoH. The curves were estimated considering epidemiological weeks as a time reference unit. To compare different curves of municipalities, the initial time point was aligned from the first case of SARS-CoV-2 infection/death in each municipality. Several curves observed presented more than one peak. To delimitate the time-span of the curves to include the main/highest peak, the observed curve was detached into distinct normal distribution using model-based clustering on parameterized finite Gaussian mixture models ^15^ . The normal distribution with the highest peak was selected and the observed curve time-span was delimited based on the amplitude of that distribution. Figure 1 shows the absolute and cumulative curves and the two parameters computed from them. The flatter the curve, the longer it takes and the slower the incremental rate until its peak. Both parameters were computed from the estimated cumulative curve using a non-linear least-square method for a five-parameter sigmoidal curve adjustment ^16^ .

To assess the flattening of curves, measured by these two parameters (incremental rate and the time to reach the peak) in each municipality, and the public policies, multiple ordinary least-square linear regressions was used. The time to reach the peak and the incremental rate were outcomes, and Stringency Index, Government Response Index, Containment and Health Index, and Economic Support Index were dependent variables representing public policies. The models were all adjusted for HDI, Crowding Rate, Demographic Density Rate, ICU Hospital Beds Rate, and Physician Rate. All statistical analyses were performed in R statistical software v.4.0.5, utilizing the ‘mclust’ library to separate the curve into distinct normal distribution and the ‘drc’ library to estimate incidence and mortality rate curve parameters.

## RESULTS

### Population

From the 1,136,681 records present in the severe acute respiratory syndrome database (SIVEP-Gripe/MoH) as of January 11, 2021, we excluded 109 patients due to lacking geographic information (country, state, city) of record origin; 2,681 due to informed age in negative number; and 202 due to informed age older than 105 years. Of the remaining 1,133,689 records, a total of 606,554 had SARS-CoV-2 infection confirmed by laboratory, clinical, clinical-epidemiological, or clinical imaging (lung X-ray/computed tomography) criteria with clinical evolution until covid-19-related death from February 20, 2020 (first confirmed infection of covid-19 in Brazilian territory) to December 31, 2020. Next, the number of deaths and the pandemic time were grouped by the municipality of residence of each registered patient. In total, 51 municipalities, the state capital, and the second city with the largest population, were included in our analysis, which means an average of two municipalities for each Brazilian state: Acre (Rio Branco, Cruzeiro do Sul); Alagoas (Maceió, Arapiraca); Amapá (Macapá); Amazonas (Manaus, Parintins); Bahia (Salvador, Feria de Santana); Ceará (Fortaleza, Caucaia); Distrito Federal (Brasília); Espírito Santo (Vitória, Vila Velha); Goiás (Goiânia, Aparecida de Goiânia); Maranhão (São Luís, Imperatriz); Mato Grosso (Cuiabá, Rondonópolis); Mato Grosso do Sul (Campo Grande, Dourados); Minas Gerais (Belo Horizonte, Uberlândia); Pará (Belém, Ananindeua); Paraíba (João Pessoa, Campina Grande); Paraná (Curitiba, Londrina); Pernambuco (Recife, Jaboatão dos Guararapes); Piauí (Teresina, Parnaíba); Rio de Janeiro (Rio de Janeiro, São Gonçalo); Rio Grande do Norte (Natal, Mossoró); Rio Grande do Sul (Porto Alegre, Caxias do Sul); Rondônia (Porto Velho, Ji-Paraná); Roraima (Boa Vista); Santa Catarina (Florianópolis, Joinville); São Paulo (São Paulo, Guarulhos); Sergipe (Aracaju, Lagarto); Tocantins (Palmas, Araguaína). Parameters of mortality rate curve could not be estimated for Rondonópolis and Parnaíba. These 51 municipalities, accounting for 28.32% of the Brazilian population, presented 261,326 records of covid-19 confirmed infection. Out of them, 55.4% are men, 34.1% declared themselves as Mixed-raced, with median age of 61 (interquartile range: 47-74) years old, 47.9% were from the Southeast region, followed by 22.2% from the Northeast region, 88% were living in the state capital, and 87.1% were living in urban areas.

### Incidence and Mortality Rate Curves and Parameter Estimation

**Figure 2:**
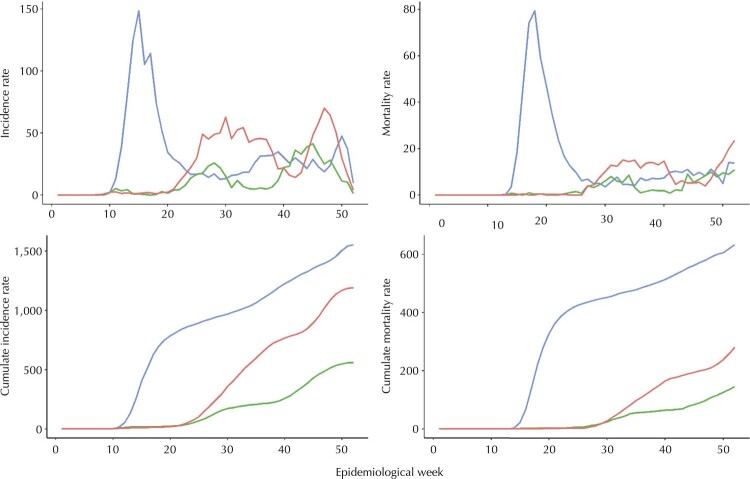
Absolute and cumulate curves of incidence and mortality rates in three municipalities by epidemiological week. Brazil, 2020.

Note: Blue, red, and green lines correspond to Manaus, Campo Grande, and Florianópolis, respectively.

The incidence and mortality rate curves indicated different patterns in the number of SARS-CoV-2 infections and covid-19-related deaths registered on SIVEP-Gripe/MoH among the 51 municipalities. Some municipalities presented a faster growth of incidence rate curve, with a high peak, followed by the mortality rate curve, indicating a fast spread of covid-19 and a high pressure on the healthcare system. On the other hand, some municipalities presented a slower incremental growth of incidence rate curve, with delayed time to reach the peak of both incidence and mortality rate curves, suggesting better control of SARS-CoV-2 infections with consequent lower mortality rates. Moreover, some municipalities presented more than one peak of infections and deaths, characterizing two different incremental rates of covid-19 curves, possibly related to the reopening process of each municipality. Figure 2 displays representative patterns (3 out of 51 municipalities) of estimated incidence and mortality rate curves (absolute and cumulative), Manaus (high incidence and mortality rates and fastened growth), Campo Grande (flatten curves, with two incremental rates well-characterized), and Florianópolis (very flatten incidence and mortality curves, with higher time to reach the peak of the curves, and two incremental rates well-characterized). These different patterns of cumulative incidence and mortality rates clearly illustrate the heterogenicity of the Brazilian covid-19 scenario in 2020. We highlight that even more diverse patterns could be observed if the data included municipalities with lower population density since they represent different realities in terms of healthcare resources and mobility rates when compared to the most densely populated cities of Brazil.

**Figure 3:**
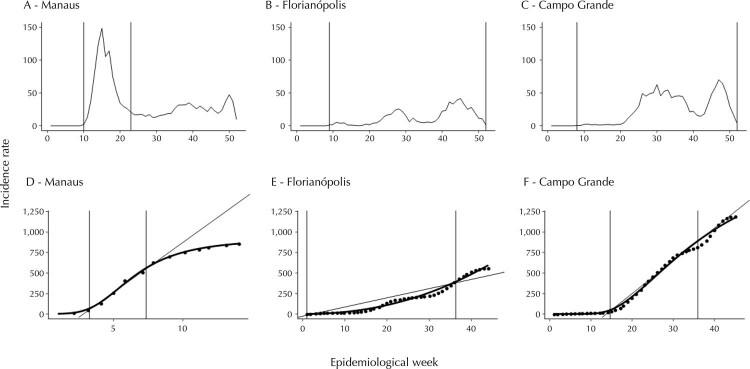
Observed incidence rate and the delimitation of time-span curve in three municipalities: Manaus (A), Florianópolis (B), and Campo Grande (C). Brazil, 2020.

Note: the first boundary corresponds to the First Week of Infection Case Notification and the last indicates the End of the Highest Peak of the Curve. Estimated (dotted line) and Observed (solid line) Cumulate Incidence Curve for Manaus [D], Florianópolis [E], and Campo Grande [F] in the region circumscribed in A, B, and C, respectively. The two vertical lines delimitate the “window of linearity,” and the inclined line is made of the region’s first and last incidence rate of the estimated curve. The tangent of the angle formed by the inclined line and the x-axis represents the slope.

The delimitation of the time-span curve to these same municipalities (i.e., Manaus, Campo Grande, and Florianópolis) is displayed in Figure 3 (A, B, and C). The first boundary corresponds to the first week of infection notification and the last one to the end of the highest peak of the curve. Figure 3 (D, E, and F) presents the cumulative incidence curves observed and estimated by the model for these same municipalities. The “window of linearity” of the curves is represented by the vertical lines, and the incremental rate of each curve was estimated by the angle formed by the inclined line and the x-axis. The time-span and incremental rate were determined for all the 51 municipalities evaluated.

### Effect of Public Policies on Incidence and Mortality Rate Curves Parameters

Table 1 presents a descriptive analysis of adjustment variables, such as socioeconomic status indexes, environmental causes of airborne transmission, healthcare assessment, and interventions adopted, i.e., public policies indexes, by the lowest (1 ^st^ quartile) and highest (4 ^th^ quartile) incidence (SIR) and mortality rate (SMR) of hospitalization by covid-19 curves for both incremental rate and time to reach the peak. These results highlight the distributions of these adjustment and intervention variables in the municipalities with low and high incremental growth rates and time to reach the peak of incidence and mortality rate of hospitalization due to covid-19. The municipalities with lower incremental rates of incidence and mortality presented higher socioeconomic statuses, i.e., HDI indexes, lower Crowding Rates, higher Stringencies, Government Responses, Containment and Health indexes, and higher ICU Hospital Beds and Physician Rates. Higher Economic Support Indexes were observed among the municipalities with a lower incremental rate of the mortality curves, but not with a lower incremental rate of the incidence curve.

**Table 1:** Descriptive analysis of Brazilian municipalities by low (1st quartile) and high (4th quartile) SIR and SMR. Brazil, 2020.

Variables	SIR ^a^		SMR ^b^	
	
	Low incidence Mean	High incidence Mean	Low mortality Mean	High mortality Mean
Slope				
HDI	0.77	0.75	0.78	0.74
HDI Education	0.7	0.69	0.71	0.68
HDI Health	0.84	0.82	0.85	0.82
HDI Income	0.78	0.75	0.79	0.72
Demographic Density Rate	295.23	298.17	377.86	244.48
Crowding Rate	20.13	34.9	19.92	39.97
ICU Hospital Beds Rate	2.97	2.77	2.84	2.37
Physician Rate	2.74	2.03	3.05	1.66
Stringency Index	62.2	54.5	67.56	66.1
Economic Support Index	4.82	10.61	8.59	8.1
Containment and Health Index ^c^	41.02	36.14	47.88	43.3
Government Response Index ^d^	36.45	32.95	42.9	38.9
Time				
HDI	0.74	0.79	0.72	0.79
HDI Education	0.67	0.72	0.66	0.72
HDI Health	0.82	0.85	0.81	0.85
HDI Income	0.73	0.81	0.71	0.81
Demographic Density Rate	344.32	275.64	296.02	263.18
Crowding Rate	38.95	18.28	39.69	18.1
ICU Hospital Beds Rate	2.71	2.94	2.57	2.78
Physician Rate	1.82	3.27	1.66	3.02
Stringency Index	54.26	62.4	65.99	65.51
Economic Support Index	1.48	10.71	3.18	14.11
Containment and Health Index ^c^	35.15	43.38	42.48	47.59
Government Response Index ^d^	30.92	39.3	37.54	43.4

^a^ SIR: standardized incidence rate of hospitalization by covid-19 curves, estimated considering the day of infection as 9 days before the date of registration at the SIVEP system. ^b^ SMR: standardized mortality rate of hospitalization by covid-19 curves. ^c^ Stringency Index augmented by health policies. ^d^ Containment and Health Index plus Economic Support Index.

In the second part of Table 1 , which refers to the time to reach the peak of incidence and mortality rate curves, we observed that 25% of municipalities with the lowest time to reach the peak presented, in general, lower socioeconomic statuses, Stringency Indexes, Government Response Indexes, Containment and Health Indexes, ICU Hospital Beds and Physician Rates, and finally, higher Crowding Rates.

The robustness analysis for the incidence rate was evaluated as a function of the uncertainty of the day of infection in relation to the day of the registration of the first symptoms of infection in SIVEP-Gripe/MoH (data not shown).

Table 2 presents the correlations between interventions, i.e., public policies, and incremental rate and time to reach the peak outcomes for the incidence and mortality rate of hospitalization by covid-19 curves, evaluated by multiple linear regression models. Considering the incidence curves, we observed a negative correlation between the Stringency Index and the incremental rate (p-value = 0.01). It means that orders to stay at home and restrictions on movement between cities, states, and countries were correlated with lower growth rates of covid-19 infections, but not with lower growth rates of covid-19-related deaths. The other public policies evaluated, i.e., Government Response Index, Containment and Health Index, and Economic Support Index, were not correlated with the incremental rate for neither incidence nor mortality rate curves. In terms of the outcome time to reach the peak of incidence curves, we identified a positive correlation with all public policies (p-values < 0.01), except for the Economic Support Index. The same results were observed for the mortality rate curves, which means that restricting people’s behavior with containment and closure policies was associated with a delay in achieving the peak of incidence and mortality rate of hospitalization by covid-19 curves. Controversially, increases in economic support were not correlated with the incremental rate of neither incidence nor mortality rate curves but presented a tendency of negative correlation with the time to reach the peak of the incidence curves (p-value = 0.06).

**Table 2:** Adjusted linear models to estimate association between covid-19 SIR and SMR and public policies in Brazilian municipalities. Brazil, 2020.

Variables	SIR ^a^	p-value	SMR ^b^	p-value
	
	Ab (95%CI)		Ab (95%CI) p-value	
Slope				
Stringency Index	**-1.17 (-2.02 to -0.31)**	**0.01**	-0.16 (-0.59 to 0.26)	0.44
Economic Support Index	0.64 (-0.19 to 1.47)	0.13	0.18 (-0.16 to 0.52)	0.3
Containment and Health Index ^c^	-0.8 (-1.8 to 0.21)	0.12	-0.15 (-0.56 to 0.26)	0.46
Government Response Index ^d^	-0.72 (-1.88 to 0.44)	0.22	-0.12 (-0.59 to 0.36)	0.62
Time				
Stringency Index	**0.35 (0.12 to 0.57)**	**< 0.01**	**0.21 (0.03 to 0.4)**	**0.02**
Economic Support Index	-0.21 (-0.42 to 0.01)	0.06	-0.07 (-0.24 to 0.09)	0.35
Containment and Health Index ^c^	**0.45 (0.21 to 0.69)**	**< 0.01**	**0.27 (0.11 to 0.44)**	**< 0.01**
Government Response Index ^d^	**0.46 (0.18 to 0.75)**	**< 0.01**	**0.3 (0.1 to 0.5)**	**< 0.01**

Ab: adjusted beta; 95%CI: 95% confidence interval.

Note: bold type indicates statistical significance.

^a^ SIR: standardized incidence rate of hospitalization by covid-19 curves, estimated considering the day of infection as 9 days before the date of registration at the SIVEP system. ^b^ SMR: standardized mortality rate of hospitalization by covid-19 curves. ^c^ Stringency Index augmented by health policies. ^d^ Containment and Health Index plus Economic Support Index.

## DISCUSSION

We investigated whether the adoption of different public policies was associated with the flattening of the covid-19-related incidence and mortality rate curves of hospitalization in Brazil.

Our evidence brings novelties and confirms results already reported. The novelty is the ineffectiveness of economic support policy in terms of “flatten the curve” strategy. Although fundamental for humanitarian, social, and economic reasons, the economic support policies seem to not have contributed to the population’s social distancing adherence since it did not affect the incidence and mortality rate of hospitalizations by covid-19 curves. Part of this unexpected result may derive from the delay in the implementation of these programs in Brazil, as well as their short duration ^17^ . Additionally, as the coefficient of the Containment and Health Index is, in general, higher than the Stringency Index, we can infer that health policies were associated with reducing the covid-19 incremental rate of incidence and deaths since the former included testing policy, facial coverings, and protection of older adults. On the other hand, our results concerning the social distancing policies (i.e., the Stringency Index) confirmed previous studies ^3 , 9^ . Using the perspective of the “flatten the curve” strategy, the Stringency Index was very effective to delay the time to reach the peaks of incidence and mortality rates of hospitalization by covid-19 curves, and reduce the incremental rate of incidence curves. On the other hand, the absence of impact of the Stringency Index on the incremental rate of mortality curves suggests that additional factors, including healthcare assessment and the population’s health condition, could be more important in terms of mortality compared to social distancing policies. Indeed, in terms of the mortality rate of hospitalization by covid-19, we observed higher incremental rates and slower times to reach the peak curves among municipalities with higher Crowding Rates and lower Physician Rates and ICU Hospital Beds Rates, which are indicators of social inequalities and limited access to healthcare resources. Precarious housing, or crowding households, was already reported as an important factor associated with SARS-CoV-2 transmission ^18 , 19^ and it is characteristic of socioeconomically less privileged populations, which were more affected by the severity of the disease ^1 , 20 , 21^ .

Among the limitations of this study, the first is that an ecological study is not the most appropriate design to verify the impact of public policies on the population since the aggregation of data may result in the loss or concealment of certain socioeconomic aspects that could be relevant to evaluating the actual impact. Furthermore, this study did not allow us to analyze causal-type relationships between public policies and the flattering of the covid-19 incidence and mortality rate curves; it only estimated the possible effects based on the correlation, even if controlled for possible confounding factors. In addition, an overlap was observed between the OxCGRT public policy indices by design. Another limitation, not from the study *per se* , but from the registry of SARS-CoV-2 infections in Brazil, was the low testing throughout 2020, especially at the beginning of the pandemic, which may have underestimated covid-19 related incidence and, even, mortality rates. Moreover, the selected municipalities do not represent the entire Brazilian reality since we only evaluated capitals and greater municipalities, excluding municipalities with low population density. In addition, we disregarded the municipalities of Rorainópolis and Santarém due to lacking information on the parameters of the curves and public policies. Finally, we were limited by the absence of population census information more recent than 2010, which forced us to use outdated measures of socioeconomic (HDI index) information, among others.

Our research evaluated which public policies were associated with the flattening of the covid-19 curves of hospitalization in Brazil. Our results indicated that the stringency of containment and closure policies decreased the incremental rate of the incidence curve, and health policies (the Stringency Index, the Containment and Health Index, and the Government Response Index) delayed the time to reach the peak of incidence and mortality rate curves. More research should be conducted to address the causality effects of specific policies on the incidence and mortality rate of hospitalization by covid-19 infections.
